# Delta-Shaped Gastroduodenostomy in Fully Laparoscopic Distal Gastrectomy: A Retrospective Study

**DOI:** 10.1097/MD.0000000000001153

**Published:** 2015-07-17

**Authors:** Tu Jian-Cheng, Zhang Bo, Fang Jian, Zhou Liang

**Affiliations:** From the Department of General Surgery, Zhangjiagang Hospital Affiliated to Soochow University, Jiangsu Province, P.R. China (TJ-C, ZB, FJ, ZL).

## Abstract

This study aims to explore the technical feasibility, safety, and clinical efficacy of delta-shaped anastomosis for digestive tract reconstruction during totally laparoscopic distal gastrectomy.

Clinical data of 24 patients who received totally laparoscopic distal gastrectomy with delta-shaped anastomosis (laparoscopic gastrectomy group, LG group) and 30 patients who received open distal gastrectomy for gastric cancer (open gastrectomy group, OG group) from April 2013 to April 2014 were retrospectively analyzed. Operation time, intraoperative blood loss, postoperative time to intestinal function recovery, postoperative pain, postoperative hospital stay, and incidence of postoperative complications (infection, obstruction, and delayed gastric emptying) were compared between these 2 groups.

Patients in both groups were discharged without marked complications. No patients who initially selected laparoscopy were converted to laparotomy. Patients in the LG group had longer operation times (175.3 ± 64.7 minutes versus 120.1 ± 43.4 minutes, *P* < 0.05), lower intraoperative blood loss (50.8 ± 25.3 mL versus 95.6 ± 20.7 mL, *P* < 0.05), faster recovery of intestinal function (1.2 ± 0.5 days versus 2.6 ± 1.0 days, *P* < 0.05), less postoperative pain (5.6 ± 0.7 versus 9.5 ± 0.3, *P* < 0.05), and shorter length of postoperative hospital stay (8.5 ± 2.2 days versus 12.2 ± 3.8 days, *P* < 0.05), compared with patients in the OG group. There were no significant differences with respect to surgical margins achieved, the number of lymph nodes retrieved or incidence of postoperative complications (infection, obstruction, and delayed gastric emptying) between the 2 groups (*P* > 0.05).

Laparoscopic reconstruction of the digestive tract through delta-shaped anastomosis appears to be safe, feasible, and associated to rapid recovery. These data argue for more wide-spread implementation of this procedure.

## INTRODUCTION

Gastric cancer constitutes the third leading cause of cancer-related death worldwide.^1^ Surgery remains the mainstay for the treatment of gastric cancer with curative intent. Encouragingly, survival following gastric cancer diagnosis has constantly improved due to better surveillance of patients at the risk of developing gastric cancer,^2^ improved adjuvant and neoadjuvant treatments,^3^ and improvements in surgical approach and techniques.^4^ However, further improvements remain necessary especially as stomach surgery is associated with significant morbidity and mortality and inadequate removal of tumor tissues.

Compared to open procedures, laparoscopic surgery is often associated with important benefits in terms of treating malignant diseases such as lower perioperative blood loss and faster patient recovery, whereas similar results are obtained with regard to tumor resection margins and oncological long-term survival. However, in stomach cancer, the value of laparoscopic surgery remains unproven. Guidelines of the Japanese Gastric Cancer Association currently recommend^5^ performing gastric cancer radical resection of the stomach with a 5 to 6 cm free margin from the tumor in combination with lymphadenectomy. The number of lymph nodes retrieved is considered as a marker for radicality of surgery and quality of care. Hence, novel surgical techniques should be noninferior with regard to the radicality of surgery and lymph node yield in addition to their clinical performance per se. Two studies have investigated the relative performance of laparoscopic versus open gastrectomy (OG),^6,7^ which supports a more widespread implementation of this operation mode. More evidence is necessary and in this respect the anastomosis techniques in particular requires further attention. An interesting development in this field was the advent of delta-shaped anastomosis, which is a modification of intracorporeal Billroth I reconstruction that was originally designed by Kanaya et al in 2002.^8^ A classic Billroth I reconstruction involves end-to-end or side-to-end anastomosis with a circular stapler; and in contrast, a delta-shaped anastomosis is intracorporeally carried out by forming a side-to-side (functional end-to-end) gastroduodenostomy by only using laparoscopic linear staplers. Thus, delta-shaped anastomosis is relatively straightforward, and the learning curve is very steep for surgeons following initial exposure to this procedure.^9^ Hence, the use of delta-shaped anastomosis in intracorporeal Billroth I reconstruction has continuously gained popularity. However, widespread implementation has been hampered because the superiority of laparoscopic technology over open procedures for treating gastric cancer in general remains unproven, and especially because the superiority of laparoscopy in combination with delta-shaped anastomosis over conventional open procedures remains untested. In this study, we aimed to address this issue by performing a retrospective analysis that compared both modalities in our cohort of gastric cancer patients. Results unequivocally reveal the superiority of laparoscopy in combination with delta-shaped anastomosis.

## MATERIALS AND METHODS

### Patients

A total of 54 patients received delta-shaped anastomosis in gastrectomy from April 2013 to April 2014. Twenty-four cases underwent laparoscopic gastrectomy (laparoscopic gastrectomy group, LG group) including 16 male and 8 female patients. Patients were 45 to 71-years old with an average age of 64-years old, and all patients were diagnosed with confirmed gastric antrum cancer and a preoperative pathological staging of T1–T3. Thirty cases underwent OG (OG group) including 21 male and 9 female patients. Patients were 51 to 82-years old with an average age of 67-years old, and all cases in this group were diagnosed with confirmed gastric antrum cancer.

### Surgical Procedures

Abdominal and pelvic cavities were examined through a gastroscope or preoperative CT scan to locate the tumors. Patients were fully anaesthetized with tracheal intubation and were placed in supine lithotomy position. The surgeon stood on the left side of the patient, the first assistant surgeon stood on the right side of the patient, and the second assistant surgeon who held the camera stood between the patient's legs. During separation of the splenogastric ligament, the surgeon positioned himself between the patient's legs. The greater omentum was severed by an ultrasound knife along the edge of the colon. The first assistant surgeon lifted the fore-stomach and omentum, and turned the stomach up in the direction of the head. The anterior lobe of the transverse mesocolon was peeled upwards; then, No. 14 lymph nodes along the inferior margin of the pancreas and surface of the head of the pancreas were removed. Subsequently, the right gastric-omentum artery was isolated from other visceral structures, and No. 6 lymph nodes were cleared away at the same time. Then, the right gastro-omental artery and left gastro-omental vein were severed and ligated, respectively. The gastroduodenal artery along the posterior wall of the duodenum was liberated, and the horizontal part of the duodenum was extensively dissected. The first assistant surgeon changed the traction, held the stomach up, and the plica gastropancreatica was subsequently exposed. Then, the splenic artery was exposed and dissected, allowing the removal of Nos. 7, 8, 9, and 11P lymph nodes. The root of the left gastric artery was then isolated and cut off with a hemoclip. No. 1 and 3 lymph nodes were cleared away from the diaphragm perpendicular to the cardia, and the lesser curvature of the stomach was severed. Nos. 5, 8, and 12 lymph nodes were removed along the common hepatic artery. The right vessel of the stomach was then ligated. The stomach was released by the first assistant surgeon, the lower edge of the liver was stretched using a tractor, and 3 to 5 cm of intestines located between the duodenum and the pylorus was carefully dissected. Subsequently, the surgeon positioned and stood between the patient's legs. Afterwards, the left gastro-omental artery and vein were isolated and cut off with a hemoclip. No. 4sb lymph node was then removed, but care was taken to leave at least 3 vasa previa vessels in situ. The surgeon returned to the left side of the patient. Then, disarticulation of gastro-duodenal resection was carried out with a linear cut stapler at approximately 2 cm down the pylorus. The resected specimen was collected and stored into a specimen bag. The specimen was removed from the incision (3 cm) of the navel or the main puncture on the left, and resection specimens were checked to make sure that no cancer remained. Then, the incision was closed, pneumoperitoneum was established, and the remnant stomach and duodenum were checked to make sure these were closed without tension. An incision (2 cm) was cut along the lesser curvature of the duodenum and another incision (3 cm) was cut along the greater curvature of the stomach. The posterior gastric wall and lateral posterior duodenal wall were sutured with a linear cutting stapler (60 mm, Johnson), and the common incision was sutured. Visual inspection was carried out to confirm that there was no bleeding. Then, the peritoneal cavity was lavaged, drainage tubes were applied according to routine procedures, and the pneumoperitoneum was closed.

Furthermore, all 30 patients in the OG group received standard radical resection of gastric cancer according to established and routine procedures.^10^

### Statistical Analyses

Data were expressed as mean ± SD and were analyzed using two-way Student *t*-test. Statistical analyses were carried out using SPSS v16.0 (SPSS, Chicago, IL). *P* < 0.05 was considered significant. Fisher exact test was used to compare the incidence of postoperative infection, obstruction, and delayed gastric emptying between the 2 groups. *P* < 0.05 was considered significant.

## RESULTS

All patients in the LG group successfully underwent laparoscopy, and no conversion to laparotomy occurred. As shown in Table 1, operation time in the LG group (175.3 ± 64.7 minutes) was longer than in the OG group (120.1 ± 43.4 minutes) (*P* < 0.05), intraoperative blood loss in the LG group (50.8 ± 25.3 mL) was lower than the OG group (95.6 ± 20.7 mL; *P* < 0.05), and postoperative time to intestinal function recovery in the LG group (1.2 ± 0.5 days) was shorter than in the OG (2.6 ± 1.0 days) (*P* < 0.05). Further, postoperative pain^11^ in the LG group (5.6 ± 0.7) was lower than in the OG group (9.5 ± 0.3) (*P* < 0.05), and length of postoperative hospital stay in the LG group (8.5 ± 2.2 days) was shorter than in the OG group (12.2 ± 3.8 days) (*P* < 0.05). There was no significant difference in surgical margins achieved or the number of lymph node dissected between the 2 groups (Table 2), with respective *P* values of 0.60 and 0.13. There was also no significant difference in the incidence of postoperative complications (infection, obstruction, and delayed gastric emptying) between the 2 groups (Table 3), with *P* values of 0.24, 0.37, and 0.20, respectively. Moreover, no fatalities occurred in both groups. Port hole infection after laparoscopic surgery occurred in 1 case in the LG group, which was successfully treated by changing the dressing. Other complications such as bleeding, anastomotic leakage or obstruction, and delayed gastric emptying did not occur.

**TABLE 1:**
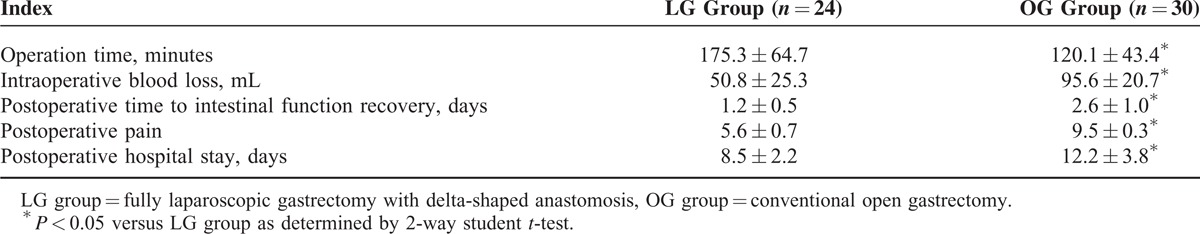
Comparison of Surgical Data Between the 2 Groups

**TABLE 2:**
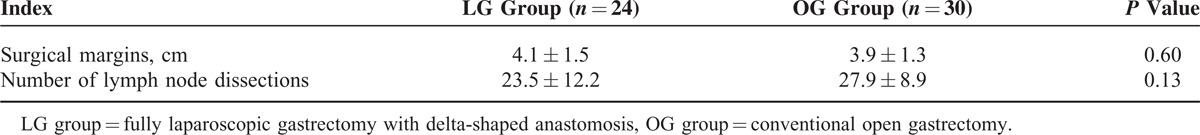
Comparison of Surgical Margins and the Number of Lymph Node Dissections in the 2 Groups

**TABLE 3:**
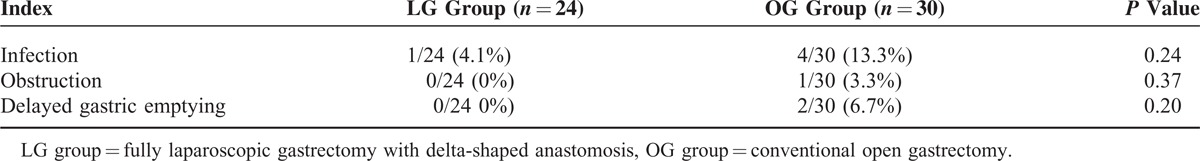
Comparison of the Incidence of Postoperative Complications of the 2 Groups

## DISCUSSION

Following the first description for laparoscopy-assisted distal gastrectomy with lymph node dissection by Kitano et al in 1994,^12^ laparoscopic gastric cancer operation has gradually evolved into a mature technology. Compared with the traditional open surgical approach, laparoscopic gastric surgery has an obvious advantage of being minimally invasive, and appears to have an equivalent, immediate, and long-term efficacy.^13^ Safety in LG is supported by 2 high-quality studies.^14,15^ There are 2 kinds of laparoscopic radical gastrectomy: totally laparoscopic surgery and laparoscopic-assisted surgery.^16^ In totally laparoscopic surgery, anastomosis is completed inside the abdominal wall and simplifies the procedure; thus, reducing operation time and surgeon workload. However, in laparoscopic-assisted gastrectomy, the incision is relatively small, especially in obese patients, yielding difficulties for the successful completion of extracorporeal gastroenteric anastomosis. Hence, this procedure is associated with significant morbidity due to postoperative complications and increased length of postoperative hospital stay. Fully laparoscopic gastroenteric anastomosis remains a challenge to surgeons and is associated with extended operation times. A possible solution was revealed by a study carried out by Kanaya et al in 2002, describing a fully laparoscopic technique to complete the gastroenteric anastomosis using a linear cutting stapler.^17^ Since then, the safety and clinical success of this specific operation has further been improved due to increased practical experience and better equipment.

Our hospital commenced performing this type of procedure in 2012.^18^ We have completed 24 cases of delta-shaped anastomosis of the remnant stomach to the duodenum. Compared to conventional circular anastomosis, the delta-shaped anastomosis is associated with a larger anastomotic area; thus, reducing the risk of anastomotic stenosis and bleeding. The conversion of our experimental procedure allows us to compare the relative performance of delta-shaped anastomosis with the classical approach, and results have been presented in this current study. In general, these support the super nature of the laparoscopic approach using delta-shaped anastomosis over the conventional open approach.

### Indications for Surgery

We introduced this novel surgical approach during the time our center was rapidly gaining experience in laparoscopic-assisted surgery. Our experience leads us to propose 3 guiding principles for selecting preoperative cases for laparoscopic surgery with delta-shaped anastomosis: the lesion is located in the gastric antrum; preoperation pathological staging is T1–T2 (we included only 1 case of preoperative pathological staging of T3, and there was no significant change in the gastric serosa) and; the anastomosis can be completed with a linear cutting stapler. This operation is an attractive option for especially relatively obese individuals who have more room in the abdominal cavity.

### Difficulties During the Operation

As described in this study, identifying lesions is one of the main difficulties during surgery, because the cancers involved were relatively in the low/early pathological stages; and localizing the lesion by probing and squeezing by manipulators may release cancer cells in the circulation. For smaller lesions, especially those in cavities, their aspect is difficult to distinguish from visual inspection of the serosa. Thus, guidance by endoscopic investigation is often necessary.^19^ Indeed, tumor localization was established through this approach in 16 cases in this present study. Following gastric resection, it is important to immediately establish an extended tumor-free margin to avoid the embarrassment of later finding out that the excision extension was insufficient. Suturing the common incision of the stomach and duodenum is another technical difficulty associated with this type of surgery. In our opinion, the part of the duodenum liberated from other tissues should be as long as possible, and it is practically advisable to have a 5 cm distance from the pylorus. When the duodenum is interrupted, the best option is to cut off the duodenum close to the pylorus and ensure a safe surgical margin at the same time to allow the common incision to be sutured without causing stenosis. The anastomosis should always be vigilantly checked to make sure it is reliable. In this study, we refrained from performing routine intensive sutures of the delta-shaped region. Based on our experience, we advise other centers that switched to this procedure to inspect the first few cases by gastroscopy.

In conclusion, although applying delta-shaped anastomosis under total laparoscopy remains somewhat experimental, the experience in our center supports a more widespread implementation of this strategy. We envision that as more experience is gained using this procedure and technology further advances, its indications would be broadened; and that totally laparoscopic radical gastrectomy would be the direction our métier would follow in the coming years.
